# A bibliometric and visual analysis of Parkinson’s disease sleep disorders: articles from 2008 to 2023

**DOI:** 10.3389/fpsyt.2024.1468568

**Published:** 2024-10-17

**Authors:** Lili Zhu, Peiyuan Zhu, Juwei Wang, Kaiwen Yan, Sheng Zhao, Yue Jiang, Huihe Zhang

**Affiliations:** ^1^ Wenzhou TCM Hospital of Zhejiang Chinese Medical University, Wenzhou, China; ^2^ Beijing University of Chinese Medicine, Beijing, China; ^3^ Wenzhou TCM Hospital of Zhejiang Chinese Medical University, Department of Graduate College, Wenzhou, China; ^4^ First Affiliated Hospital of Wenzhou Medical University, Department of Acupuncture, Wenzhou, China

**Keywords:** sleep disorders, Parkinson’s disease, bibliometrics, visual analysis, CiteSpace, VOSviewer

## Abstract

**Objective:**

Sleep disorder is a common non-motor symptom (NMS) of Parkinson’s disease. However, the global research focus on Parkinson’s sleep-related disorders (PDSDs) and future trends remains unclear. Currently, there is no bibliometric analysis of PDSDs. We aim to fill this gap, determine the status of current research, and predict future research hotspots.

**Methods:**

We selected 1490 publications from the Web of Science Core Collection (WoSCC) database from 2008 to 2023. Based on CiteSpace and VOSviewer, the analysis was performed from the perspectives of the trend in the number of annual publications, countries, institutions, authors, journals, and co-citations.

**Results:**

A total of 1490 publications from 590 authors from 409 institutions in 77 countries are included. The United States, China, and the United Kingdom are the leading countries. University College London (UCL) is the most prolific institution. Harvard University is the key for cooperation among institutions. Chaudhuri Kallol Ray is a leader in this field. “Movement Disorders” is the most influential journal. “A systematic review of the literature on disorders of sleep and wakefulness in Parkinson’s disease from 2005 to 2015” is the publication with the highest co-citation intensity.

**Conclusion:**

The total volume of publications on PDSDs is on the rise, entering a relatively high-yield stage in 2020. The COVID-19 pandemic and the emergence of new keywords may be the reasons behind this phenomenon. “quality of life” and “circadian rhythm” are the mainstream topics of PDSD research. Daytime sleepiness is the PDSD subtype that has received the most attention. Sleep quality, biomarkers, and neurodegeneration are likely to become future research hotspots.

## Introduction

Sleep disorders are common nonmotor symptoms (NMSs) of Parkinson’s disease, and they become more common as the disease progresses (1). Both pathological changes in Parkinson’s disease and the use of dopamine drugs can lead to a higher than expected incidence of sleep disorders (2). Studies have shown that approximately two-thirds of Parkinson’s disease patients will develop sleep disorders (3). The subtypes mainly included insomnia, daytime sleepiness with sleep attacks, restless legs syndrome (RLS), REM sleep behavior disorder (RBD), sleep-related breathing disorder (SRBD) and circadian rhythm sleep-wake disorder (CRSWD) (4). A long-term lack of sleep severely reduces the quality of life of patients (5) and promotes neurodegeneration by exacerbating neuroinflammation (6), which accelerates the progression of Parkinson’s disease. In addition, sleep disorders predict other NMSs, such as fatigue, cognitive decline, and depressive symptoms (7).

There is no shortage of high-quality reviews in the field of Parkinson’s disease sleep disorders (PDSDs), but quantitative and qualitative analyses of the literature are lacking. Therefore, it is necessary to perform bibliometric analysis. This method was proposed by Alan Pritchard in the 1960s (8). Compared with traditional reviews, bibliometric analysis processes a large amount of literature data, summarizes the current research status and hot topics in a specific field, and provides guidance for predicting future research trends (9, 10). This method has become an important means to evaluate the scientific research output of countries, academic institutions, individual authors, and journals. Today, it can be performed with the help of software that can generate visualization graphics. The most commonly used software programs are CiteSpace and VOSviewer (11). CiteSpace was developed by Professor Chaomei Chen from Drexel University in the United States (12). VOSviewer is distributed by Nees Jan van Eck and Ludo Walterman (13).

From the perspective of bibliometrics, this study uses CiteSpace and VOSviewer to analyze the literature in the field of PDSDs, providing a quick guide for new researchers. This includes identifying leading countries, institutions, journals, and the most influential authors and literature in this field. Additionally, the statistics of high-frequency keywords highlight the current hot topics, providing insights for future research trends.

## Methods

### Data sources and retrieval strategy

We used the following query: TS=(“Parkinson” AND (“insomnia” OR “restless legs syndrome” OR “excessive daytime sleepiness” OR (“sleep” AND (“quality” OR “disturbance” OR “disorders”)))). The publications were retrieved from the Web of Science Core Collection (WoSCC). The publication types were set as articles and review articles, the time span was from 2008 to 2023, the language was English, and the index formats included Science Citation Index Expanded (SCI-EXPANDED), Current Chemical Reactions (CCR-EXPANDED), and Index Chemicus (IC).

### Data extraction and processing methods

Our search was completed on February 7, 2024. A total of 3665 publications were retrieved. We appropriately read each publication, selected objects that met our requirements, and exported them as plain text files. Screening was done by a two-person team, and the raw data was reviewed by all members. Publications are screened based on the following inclusion and exclusion criteria:

### Inclusion criteria:

1. The research topic of the publication is PDSDs.

2. The research object of the publication is not PDSDs directly, but involves the main pathogenic mechanism, precursor diseases, signal pathways, etc.

3. Only part of the publication addresses PDSDs, but the content is large, covering at least one chapter.

### Exclusion criteria:

1. The research topic and content of the publication are unrelated to PDSDs.

2. Although part of the publication covers PDSDs, the content is minimal, typically consisting of a few quotations or statements, without in-depth discussion.

**Figure 1:**
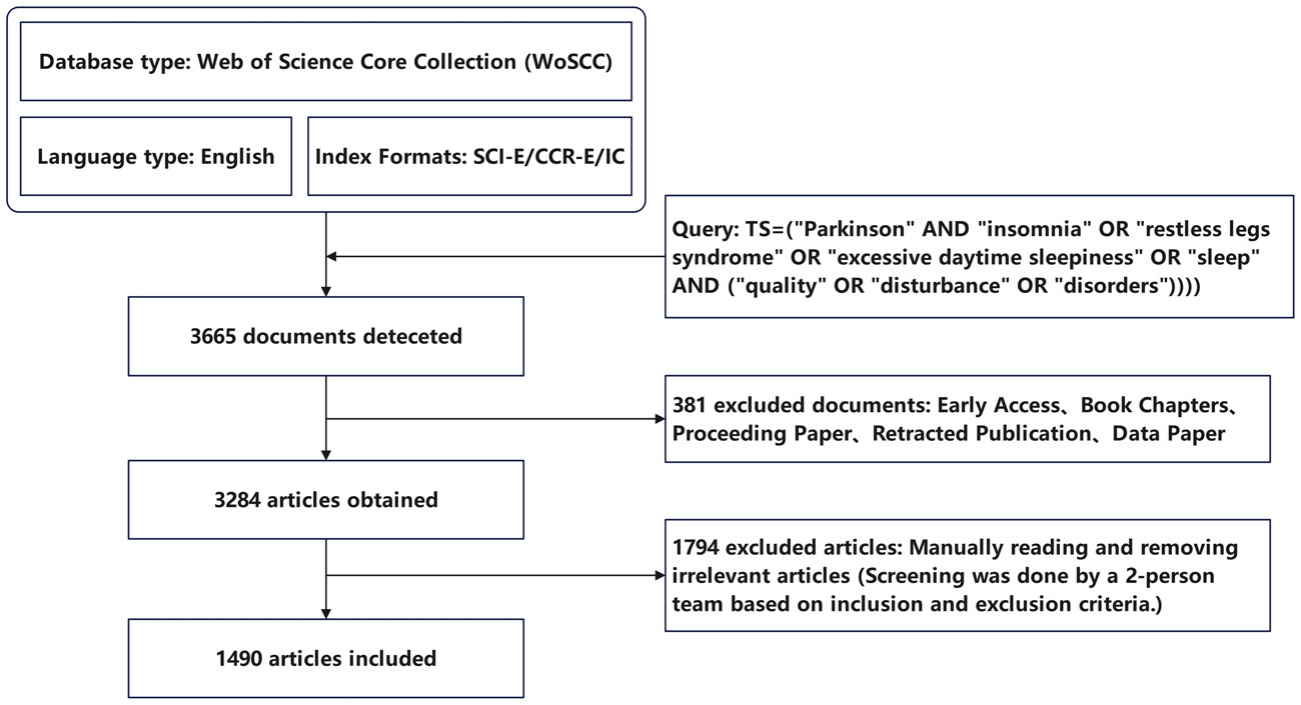
Data extraction process.

We finally obtained 1490 records that met the requirements. This process is shown in **Figure 1**. These data did not involve the privacy of patients and therefore did not require ethical review and could directly enter the data analysis link. We mainly used CiteSpace (v6.2 r3), and VOSviewer1.6.19 was used to supplement the journal publication volume and citation statistics.

### Software parameters and results output

CiteSpace operates with the following parameters: The time span is 2008-2023, and “Years Per Slice” is set to 1; “Selection Criteria” remains its default parameter; Minimum Spanning Tree(MST) algorithm is used in the parts of country, institution and keywords section to provide a more clear co-occurrence path. The author section employs the Pathfinder algorithm to enhance core author collaboration visibility and is analyzed based on Lotka’s Law; Bold highlights have been applied to signify strong links between all nodes; The keyword clustering is visualized in the format of Landscape map; The future hot spot prediction is based on burstness algorithm and subgroup analysis integrating both publication burst stage and keyword burst stage. Citation paths between journal clusters are illustrated through dual-map overlays, based on VOS rule. VOSviewer uses the default settings to generate output of journal rankings.

### Interpretation of main parameters

#### Centrality

Centrality is used to assess the significance of a node within a network. In general, nodes with higher Centrality values indicate a greater number of connections to other nodes.

#### Z score

Z score is a Dual-map overlays parameter with a trend similar to frequency (14). Both are used to label the citation intensity between journal clusters. Generally, higher values typically result in thicker reference paths, symbolizing a higher number of citations.

## Results

### Annual statistics of article publications

**Figure 2:**
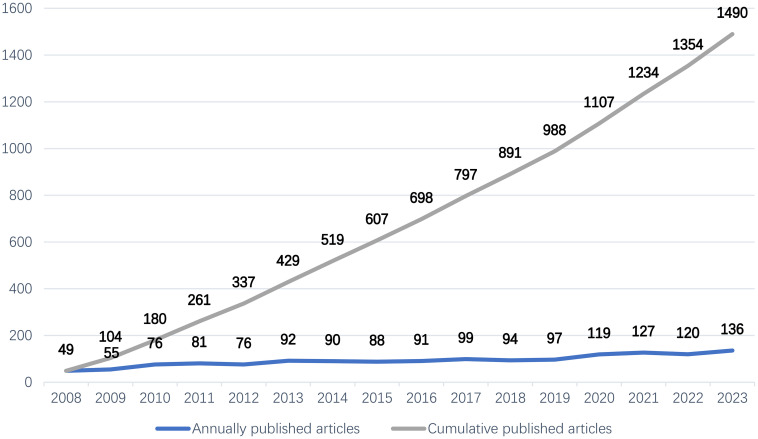
The number of annually published articles and the cumulative number of published articles.

We included 1490 publications from the WoSCC as a sample and plotted **Figure 2** to show the annual trend of publications, with the number of published papers on the vertical axis and the year on the horizontal axis. The results reveal that the total number of publications on PDSD patients is increasing. From the perspective of the annual volume of publications, we divided publications into three stages: 1. During the 2008-2009 period, the annual volume of publications was relatively small. 2. During the 2010-2019 period, the publication volume increased slowly, and the overall volume was relatively stable. 3. The annual volume of publications since 2020 has exceeded 100, entering a relatively high-yield period.

### Country and international collaborative work analyses

**Figure 3:**
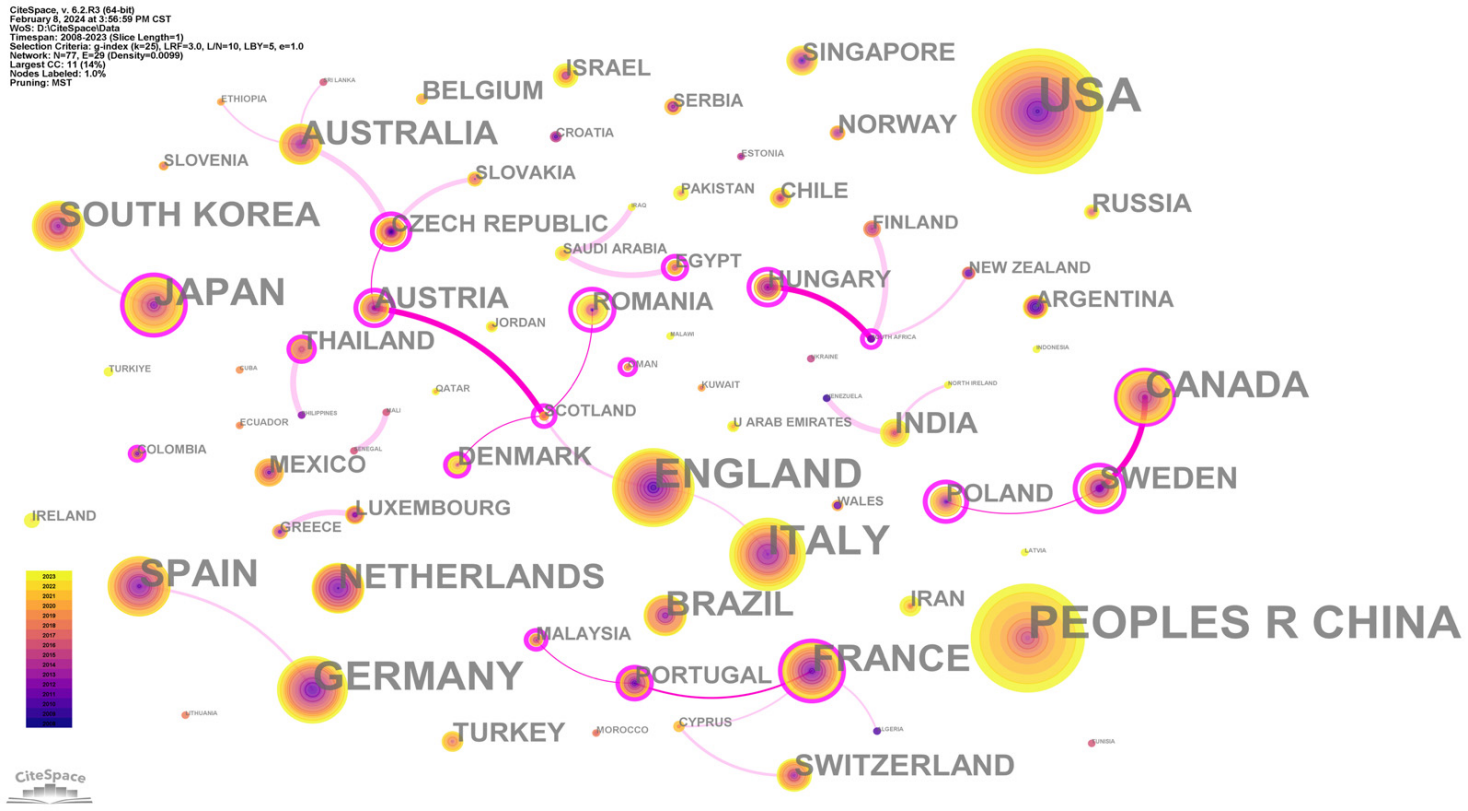
The main collaboration network of countries/regions.

Analysis reveals that a total of 77 countries have contributed to this field. We compiled the top 10 countries in **Table 1**. More than half of the publications were from the top three countries. The United States had the largest number of publications (349, 23.4%), followed by China (274, 18.3%) and the United Kingdom (152, 10.2%). The statistics for the United Kingdom include England(140), Scotland(5), Ireland(4) and Wales(3). Most of the countries on the list are in Europe. Based on the minimum spanning tree (MST) algorithm, we produced a visualization map (**Figure 3**) to show the main cooperative relationships among countries. The thickness of the connection line represents the intensity of cooperation, and the critical cooperation path is highlighted. Based on a comprehensive consideration, we consider the following combinations to be close partners: Hungary and South Africa, Canada and Sweden, and Austria and Scotland.

**Table 1:** Top 10 countries with the highest productivity.

Rank	Countries	Publications	Regions
**1**	United States	349	North America
**2**	Peoples’ Republic of China	274	East Asia
**3**	United Kingdom	152	Europe
**4**	Italy	135	Europe
**5**	Germany	126	Europe
**6**	Spain	99	Europe
**7**	Japan	96	East Asia
**8**	France	90	Europe
**9**	Canada	82	North America
**10**	Netherlands	61	Europe

### Institution analysis

**Figure 4:**
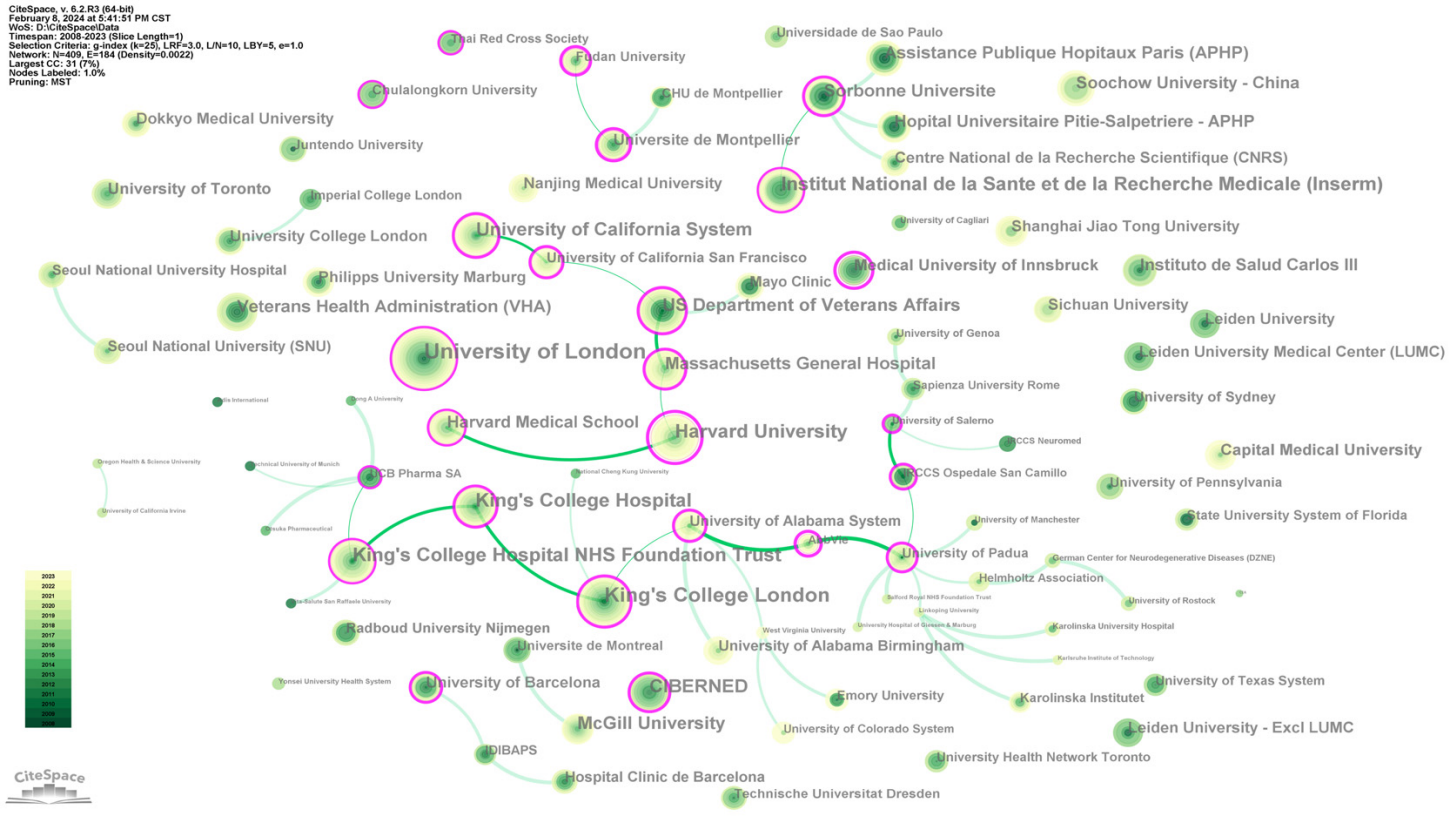
The main collaboration network of institutions.

A total of 409 institutions have contributed to this field. Based on the MST algorithm, we constructed a map of major interinstitutional collaboration networks (**Figure 4**) and listed the 10 most productive institutions (**Table 2**). The results show that University College London (UCL) was the most prolific institution, with a total of 81 papers published. In addition, the centrality value of Harvard University reached 0.93, indicating that it plays a core role in institutional cooperation and has a very high voice in institutional cooperation. Interestingly, in the country analysis, the United States, China, and the United Kingdom were the leading countries. However, institutions from the United Kingdom play a dominant role in the list, while no institution from China is on the list. This finding indicates that British publications tend to be concentrated in a few powerful institutions, while Chinese publications tend to be scattered; the distribution of American journals is somewhere in between. In addition, the statistical data of the UK institutions and the national data are somewhat different; this is a normal phenomenon, and we will explain it in the Discussion section.

**Table 2:** The top 10 institutions with the most publications.

Rank	Institutions	Publications	Centrality	Country
1	University College London (UCL)	81	0.10	United Kingdom
2	King’s College London	60	0.30	United Kingdom
3	King’s College Hospital NHS Foundation Trust	49	0.19	United Kingdom
4	Institut National de la Sante et de la Recherche Medicale	48	0.12	France
5	Harvard University	48	0.93	United States
6	King’s College Hospital	46	0.29	United Kingdom
7	University of California System	44	0.21	United States
8	CIBERNED	40	0.26	Spain
9	McGill University	37	0.05	Canada
10	US Department of Veterans Affairs	35	0.32	United States

### Analysis of contributing authors

**Figure 5:**
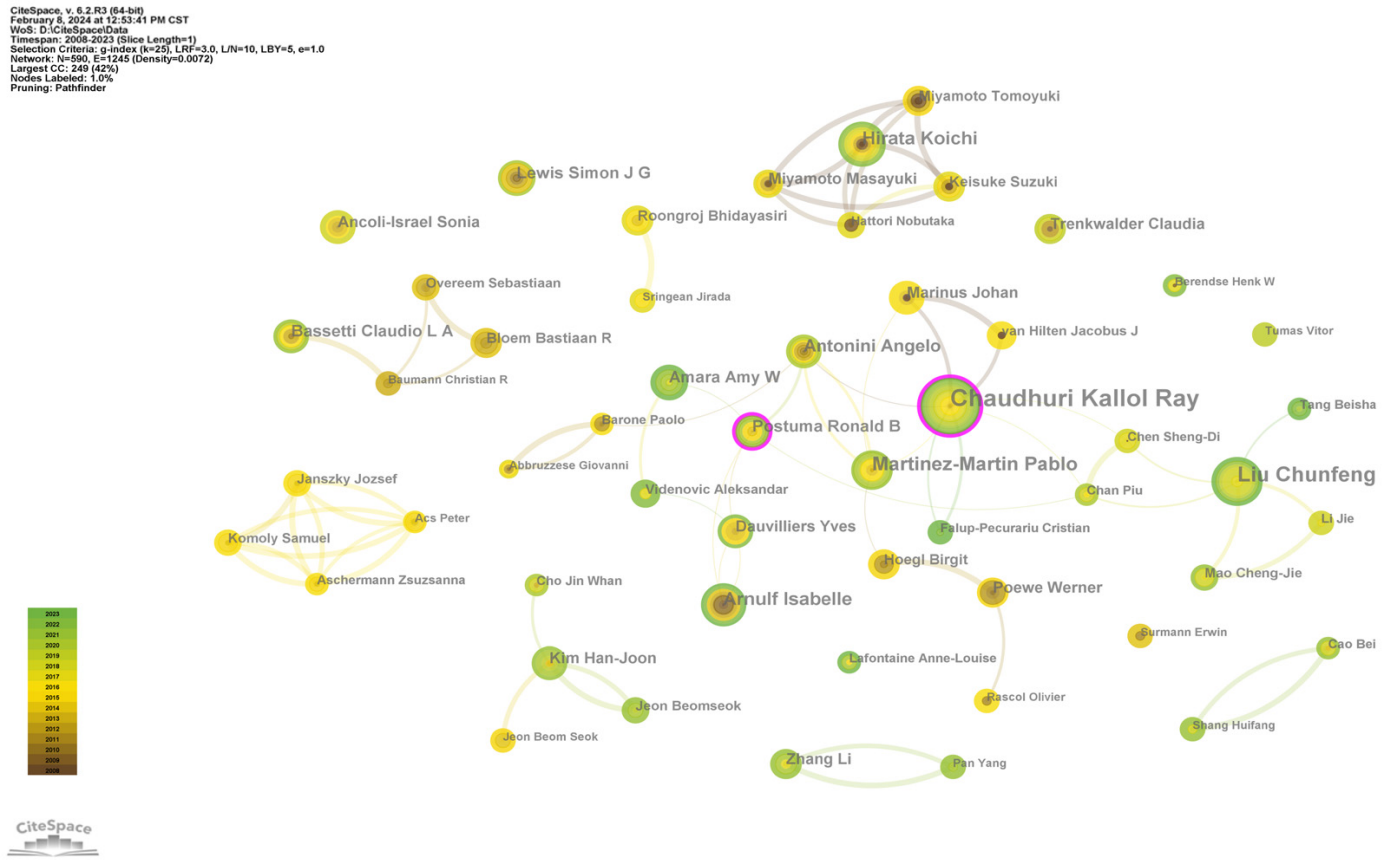
The collaboration network of the core authors.

A total of 590 authors were included in the analysis. Among all the authors, the median number of publications was 2, and the number of authors with three or more publications was 151. From the perspective of Lotka’s Law, a core group of authors will contribute the most to productivity (15). Price set the condition for becoming a core author such that the number of publications n=0.749 $\sqrt{N\max}$ ,where Nmax is the number of publications by the most prolific author (16). The 10 authors with the highest productivity are listed in **Table 3**. At the top of the list is Chaudhuri Kallol Ray, who has published a total of 44 papers. Therefore, the threshold for becoming a core author is 5 papers. According to the statistics, 52 core authors published a total of 464 papers, accounting for 31.1% of the total publications. This finding shows that the PDSD field has not yet formed a stable core group of authors and is still in the development stage. Based on the Pathfinder algorithm and the pruning of redundant branches, we present the collaboration network of these core authors in **Figure 5**.

**Table 3:** The top 10 most productive authors.

Rank	Authors	Papers	Principal affiliations
1	Chaudhuri Kallol Ray	44	King’s College London
2	Liu Chunfeng	24	Soochow University
3	Martinez-Martin Pablo	18	CIBERNED
4	Arnulf Isabelle	16	Pierre and Marie Curie University
5	Hirata Koichi	16	Dokkyo Medical University
6	Antonini Angelo	13	University of Milan
7	Bassetti Claudio LA	12	University Hospital Zurich
8	Amara Amy W	12	The University of Alabama at Birmingham
9	Lewis Simon JG	12	The University of Sydney
10	Postuma Ronald B	11	McGill University

### Journal analysis

A total of 336 journals published articles. **Table 4** shows the top ten journals with the highest productivity and their citations. The results show that “Parkinsonism & Related Disorders” was the most productive journal. However, considering that “Movement Disorders” has a greater number of citations and a higher impact factor, we list it as the most influential journal in this field.

**Table 4:** The top 10 most productive journals.

Rank	Full journal title	Papers	Citations	IF2023	WOS categories
1	Parkinsonism & Related Disorders	104	3395	4.1	Clinical neurology
2	Movement Disorders	91	8541	8.6	Clinical neurology
3	Sleep Medicine	64	1825	4.8	Clinical neurology
4	Frontiers in Neurology	46	656	3.4	Clinical neurology
5	Journal of Neurology	41	1751	6.0	Clinical neurology
6	Journal of Parkinson’s Disease	40	566	5.2	Neurosciences
7	Parkinson’s Disease	40	699	3.2	Clinical neurology
8	Journal of the Neurological Sciences	38	1076	4.4	Clinical neurology
9	Neurological Sciences	33	602	3.3	Clinical neurology
10	PLOS One	29	880	3.7	Multidisciplinary sciences

**Figure 6:**
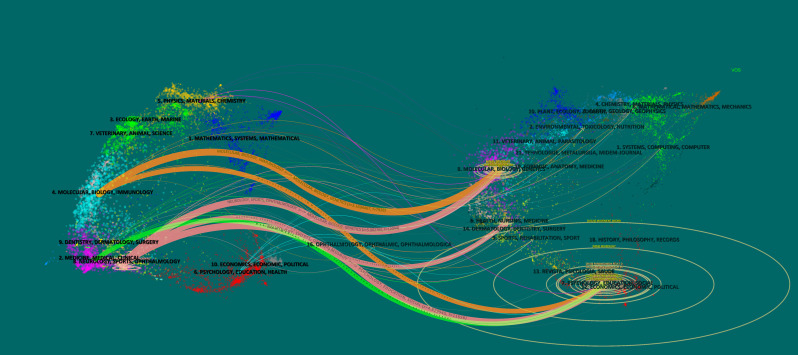
Dual-map overlays of sleep disorders in Parkinson’s disease.

A dual overlay map was created (**Figure 6**). The clustering of cited journals is based on the VOS rule, and this map can show detailed interjournal citation paths (17). According to the results, PDSD citing journals were mainly from three major fields, and the cited journals were mainly from two major fields. We collated the data of these fields/clustering and each citation path into **Table 5**. The core citation paths are as follows: journals from the fields of “neurology, sports, ophthalmology” cite journals from the fields of “molecular, biology, genetics”. There were 12,044 citations and a Z score of 5.002765.

**Table 5:** Main citation path statistics of dual-map overlays.

Rank	Citing journal cluster	Cited journal cluster	Citations	Z score
1	Neurology, sports, ophthalmology	Molecular, biology, genetics	12044	5.002765
2	Neurology, sports, ophthalmology	Psychology, education, social	11656	4.829945
3	Molecular, biology, immunology	Molecular, biology, genetics	7928	3.1694467
4	Molecular, biology, immunology	Psychology, education, social	5032	1.3795315
5	Medicine, medical, clinical	Psychology, education, social	4588	1.6817684

### Analysis of co-citations

We conducted co-citation analysis on the following three aspects: cited articles, cited authors, and cited journals. Co-citation analysis was proposed by the American scholar Henry Small in 1973 (18). This method can effectively identify high-quality cited objects that cross domains (19) to supplement our analysis above from a new perspective. This analysis was completed using CiteSpace, which aimed to screen the top 3 members with co-citation strengths in each aspect by constructing a co-citation network within the sample. The results are plotted in **Table 6**.

**Table 6:** Top 3 members in terms of co-citation strength: Cited articles, cited authors, and cited journals.

Part					
**Cited** **Articles**	Rank	Title		Author	Co-Citation Strength
	1	A systematic review of the literature on disorders of sleep and wakefulness in Parkinson’s disease from 2005 to 2015		Chahine Lana M	83
	2	The PRIAMO study: a multicenter assessment of nonmotor symptoms and their impact on quality of life in Parkinson’s disease		Paolo Barone	58
	3	Rotigotine effects on early morning motor function and sleep in Parkinson’s disease: a double‐blind, randomized, placebo‐controlled study (RECOVER)		Claudia Trenkwalder	58
**Cited** **Authors**	Rank	Name	H-index	WOS ID	Co-Citation Strength
	1	Chaudhuri Kallol Ray	81	FZT-9260-2022	569
	2	Postuma Ronald B.	70	ABE-7465-2020	456
	3	Braak Heiko	105	EML-3451-2022	326
**Cited** **Journal**	Rank	Name			Co-Citation Strength
	1	MOVEMENT DISORD			1420
	2	NEUROLOGY			1267
	3	PARKINSONISM & RELATED DISORDERS			1072

### Analysis of keywords

**Figure 7:**
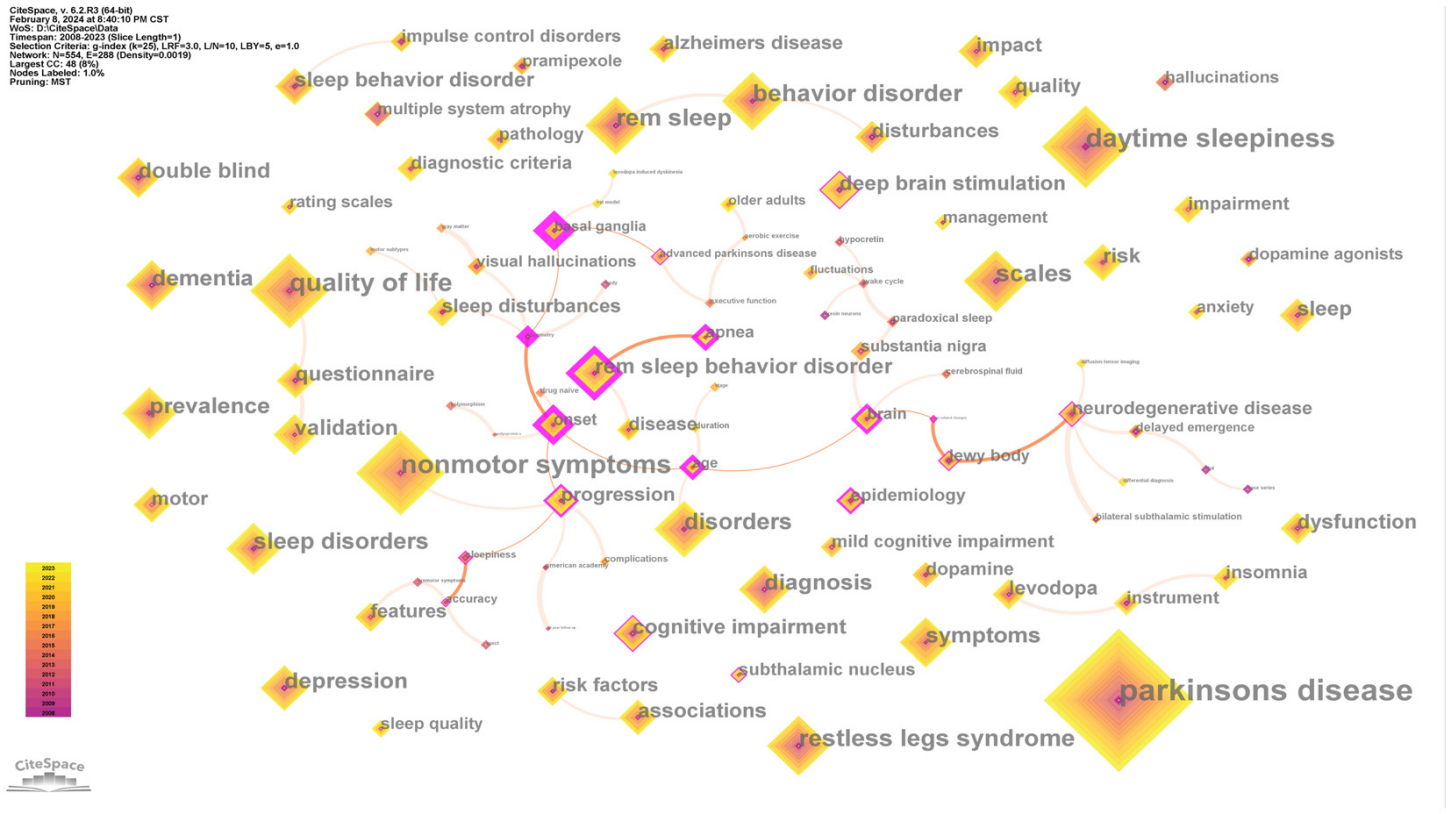
The main collaboration network of keywords.

A total of 554 keywords were extracted from this analysis. **Table 7** lists the top 10 keywords by frequency. In terms of the study subjects, a considerable portion of the literature involved comprehensive analysis of the NMSs of Parkinson’s disease patients. This process usually included studies on sleep, and therefore, NMSs rank second on the list. From the perspective of Parkinson’s disease subtypes, daytime sleepiness has received the greatest degree of attention. In addition, an analysis diagram of the main co-occurrence relationships of keywords based on the MST algorithm was drawn, as shown in **Figure 7**.

**Table 7:** The top 10 keywords by frequency.

Rank	Keywords	Frequency
1	Parkinson’s Disease	1052
2	Nonmotor Symptoms	423
3	Daytime Sleepiness	406
4	Quality of Life	326
5	Scales	238
6	Rem Sleep	226
7	Restless Legs Syndrome	223
8	Behavior Disorder	209
9	Disorders	199
10	Sleep Disorders	176

**Figure 8:**
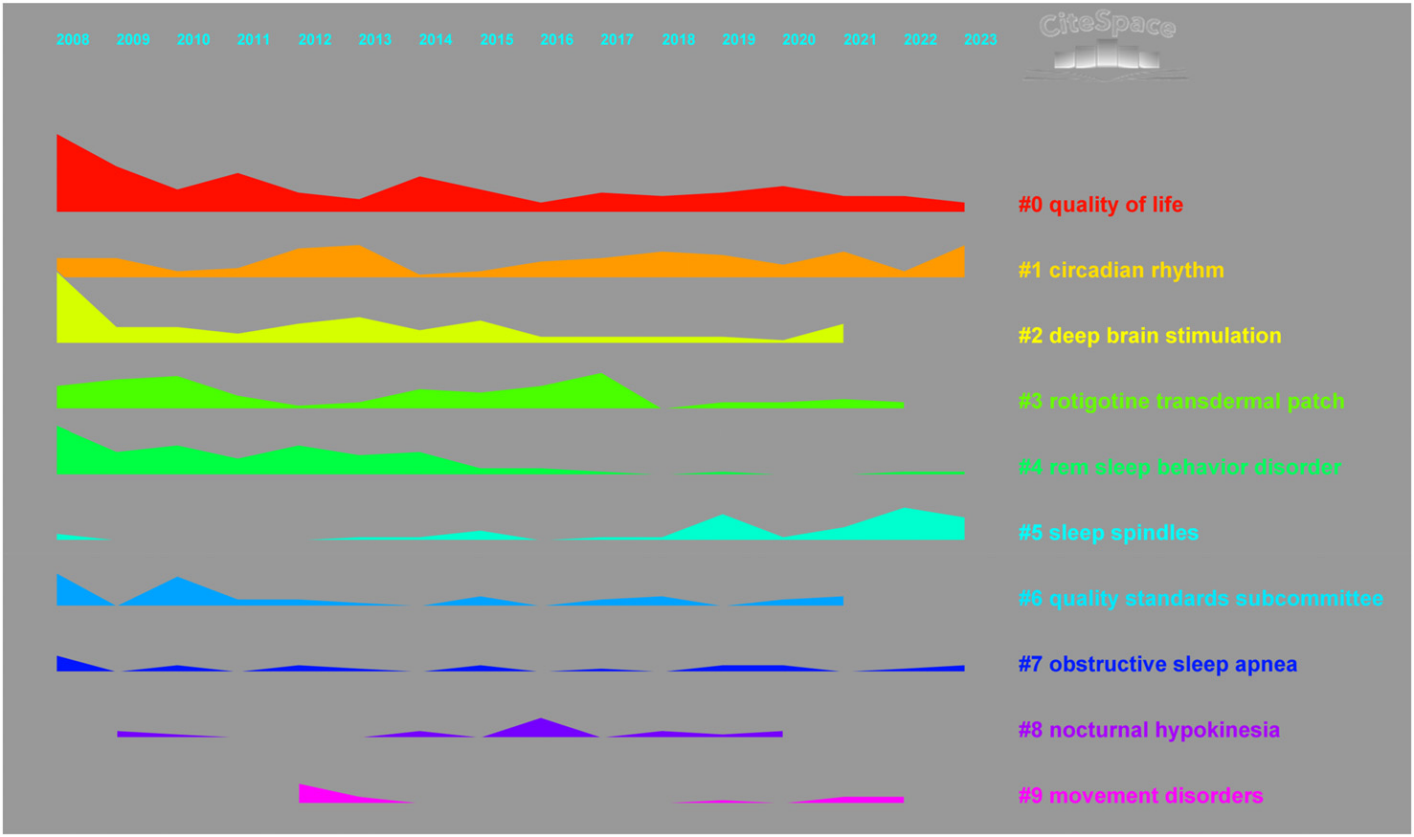
Landscape map of keyword clusters.

We clustered the keywords and obtained a total of 9 tags. **Figure 8** was obtained by drawing all the labels in the form of a Landscape map. Considering the size and duration of the clustering labels, we list “quality of life” and “circadian rhythm” as the most mainstream PDSD topics.

**Figure 9:**
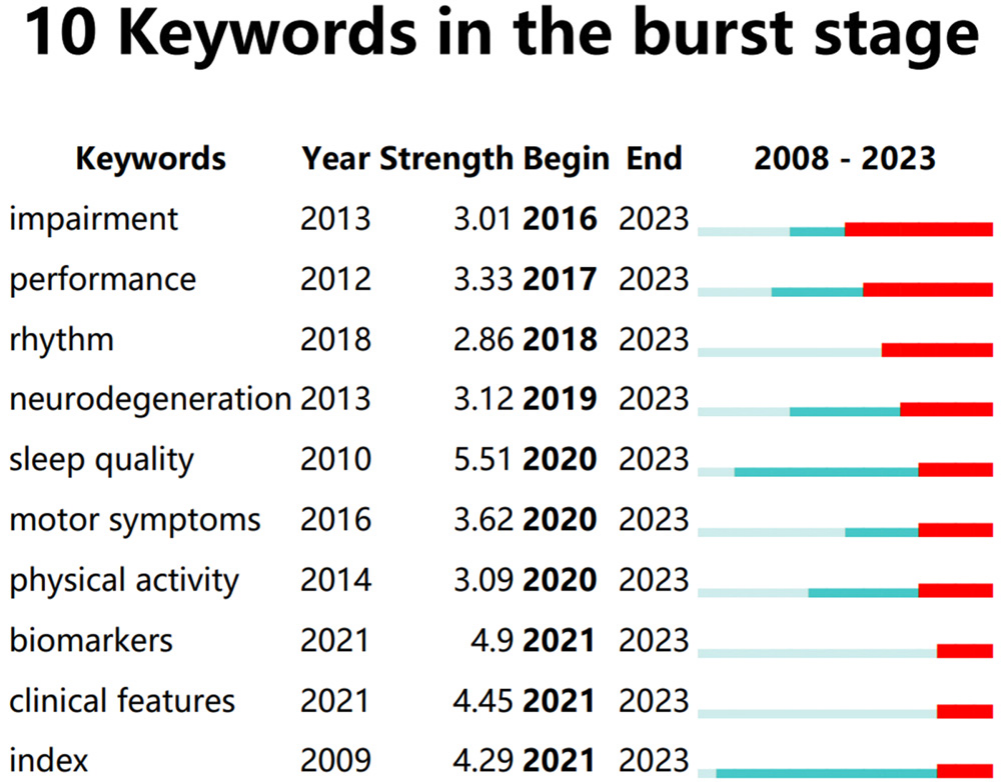
10 Keywords in the burst stage.

The burstness algorithm is used to screen keywords whose popularity has risen significantly during a certain period of time. If some keywords are still in the burst period, this reflects that they may be hot topics in the future. A total of 53 burst keywords were generated. We extracted 10 keywords that are still in the burst stage, as shown in **Figure 9**, to provide some assistance for future research and prediction.

## Discussion

### The trend of literature publications

A total of 1490 publications from 590 authors from 409 institutions in 77 countries are included in this analysis. The total number of publications on PDSDs is on the rise, entering a relatively high-yield stage in 2020. We analyze the possible reasons behind this phenomenon as follows:

1. Regarding the impact of the novel coronavirus (COVID-19), our search found that the numbers of articles related to COVID-19 in the past four years were 2, 3, 5, and 4 in the samples. On the one hand, these relevant articles contribute partially to this increment. On the other hand, since COVID-19 is a global pandemic, by the end of 2022, more than 445 million cases had been reported worldwide (20). A significant number of these cases involved individuals with Parkinson’s disease. COVID-19 can increase the occurrence of sleep disorders in Parkinson’s disease patients (21). Therefore, more case groups that are more easily noticed by researchers may be generated. This potential association is deemed a plausible explanation for this trend.

2. The influence of some burst keywords, i.e., keywords that are still in the burst stage, represents potential hotspots. There are 6 keywords that burst in 2020 and after. The aggregate frequencies of their publications from 2020 to 2023 are as follows: sleep quality (27), motor symptoms (15), index (13), biomarkers (11), clinical features (10), and physical activity (8). We consider these themes to be potential factors leading to the high yields in 2020.

Considering that the PDSDs field has not yet formed a stable core group of authors, and combined with the above potential factors, we believe the field has broad prospects and may soon experience rapid development. More productive researchers and high-quality research output are expected to emerge.

### Characteristics of literature producers

We conducted this study from the perspectives of countries, institutions, authors, journals and co-citations and summarized the characteristics as follows.

The United States, China, and the United Kingdom are the leading countries. University College London (UCL) is the most prolific institution. When King’s College Hospital and related institutions are combined, they become the most productive system. In addition, the number of studies by the University College London (UCL) and the King’s College Hospital system exceeds the total number of studies in the UK. This finding indicates that there should be a strong co-authorship relationship among these institutions, with the generated double counts leading to this result. Judging from the map generated by the MST algorithm, the King’s College Hospital system has a close cooperative relationship within the system. This finding confirmed our hypothesis. Interestingly, the University of London is more inclined not to choose specific partners for cooperation. The Chinese research model seems unhealthy. On one hand, there is a lack of productive institutions; on the other, there is a lack of international cooperation. In the future, Chinese researchers can try to promote more in-depth research and strengthen cooperation with foreign institutions.

Chaudhuri Kallol Ray is the author with the highest number of publications and has the highest co-citation intensity among authors; thus, he is a leader in this field. We also estimated the size of the core author group in the PDSD field, and the results show that this field is still in the development stage. From the perspective of journals, “Parkinsonism & Related Disorders” is the most productive journal. “Movement Disorders” is the most cited journal and the journal with the highest co-citation intensity, which fully proves its strong influence in this field. In addition, the core citation path between journals is “neurology, sports, ophthalmology → molecular, biology, genetics”.

“A systematic review of the literature on disorders of sleep and wakefulness in Parkinson’s disease from 2005 to 2015” is the publication with the highest co-citation intensity, and it was published by Elsevier in 2017 by Chahine Lana M (22). This was a systematic review based on the Preferred Reporting Items for Systematic Reviews and Meta-Analyses (PRISMA) guidelines, and it described the epidemiology, etiology, clinical implications, associated features, evaluation measures, and management of these disorders. Compared with direct citation, co-citation analysis can more accurately reflect the research frontier and effectively identify high-quality literature. Therefore, we recommend reading this article to quickly understand the diagnostic criteria and treatment options for PDSDs (23).

### Research hotspots and trends

**Figure 10:**
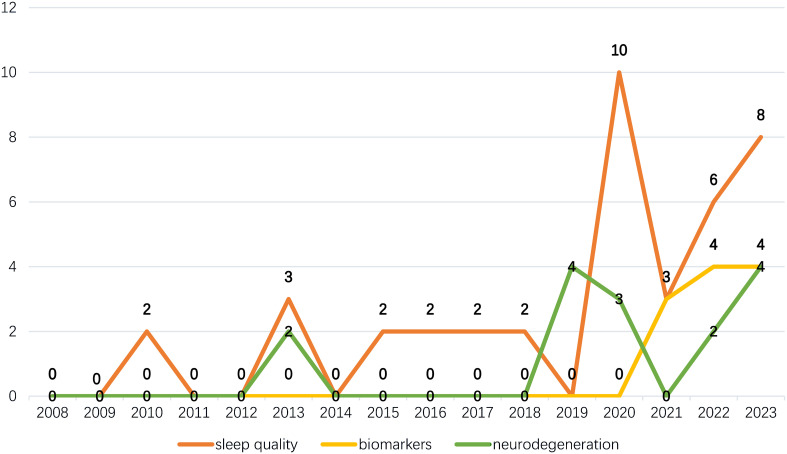
The number of annually published articles containing potentially hot keywords.

We analyzed the keywords. Daytime sleepiness is the PDSD subtype that has received the most attention. The results of the cluster analysis show that “quality of life” and “circadian rhythm” are the mainstream PDSD topics. Trends on the Landscape map indicate that these two topics span the entire period without interruptions or significant declines. From the perspective of burst keywords, a total of 10 entries are in the burst stage. Among them, 3 keywords have further increased in popularity in recently: sleep quality, biomarkers, and neurodegeneration. An illustration, as depicted in **Figure 10**, presents the annual publication volume of articles incorporating these topics. Utilizing this data, we performed a subgroup analysis of their samples over the past four years to forecast potential future areas of interest.

Sleep quality was the most comprehensive topic, involving a total of 27 publications; the research type was mainly clinical studies, with 18 publications in total. From a citation perspective, people are more concerned about the possibility of wearable devices monitoring the nocturnal movement symptoms of Parkinson’s disease (24, 25). The improvement in the sleep quality of Parkinson’s disease patients through exercise therapy is also a novel perspective (26, 27). In addition, the connection between RBD and cognitive impairment in Parkinson’s disease patients may receive increased attention in the future (28).

Biomarkers are a new keyword that officially appeared in 2021, involving a total of 11 publications. This keywork provides a new perspective for the prediction and diagnosis of sleep disorders. For example, olfactory dysfunction could be used as a predictor of Parkinson’s early-stage RBD (29). Cerebrospinal fluid (CSF) soluble TREM2 protein (sTREM2) is associated with the pathogenesis of Parkinson’s disease and helps predict sleep disorders in Parkinson’s disease patients (30). Monitoring serum microRNAs in RBD patients is expected to predict the pathogenesis of Parkinson’s disease (31). In short, researchers seem to be more interested in detecting biomarkers in patients with RBD, which could aid in the early detection and treatment of Parkinson’s disease.

The neurodegeneration sample was the smallest, involving a total of 9 publications. Most of these studies lack common themes and instead include neurodegeneration-related discussions. We consider this finding to be the result of gradual developments in the PDSD field. As pathological studies of neurodegeneration gradually improve, researchers are becoming more willing to include this perspective in their papers. For example, one study inserted an A53T mutation based on the alpha-synuclein gene (SNCA) to create a novel mouse model of precursor Parkinson’s disease that exhibited RBD-like behavior and hypothermia without motor symptoms (32). Another study, based on quantitative sleep EEG, indicated that lower sleep spindle density was a predictor of lower cognitive scores in PD patients with RBD (33). The disease course map is a novel perspective. A study mapped the disease course of PD patients using this method, demonstrating that the disease course of PD patients with RBD progresses faster than that of patients without RBD from a visual perspective. This is especially evident in isolated RBD patients (34).

From the results of subgroup analysis, RBD remains a significant research focus. Although our Landscape map analysis suggests that RBD itself is not currently a hot topic, many studies still use it as a basis. Therefore, it continues to be an important underlying theme. Future research will likely see an increase in studies based on these findings. Moreover, despite increasing research on PDSDs, the early diagnosis and treatment during the prodromal stage remain critical challenges in Parkinson’s disease management. Our keyword analysis predicts that more pathologic pathways/mechanisms of PDSDs will be identified, leading to the discovery of more biomarkers. These could enhance monitoring the effectiveness of RBD treatments.

## Strengths and limitations

This study is based on the bibliometric and visualization analysis of PDSDs, filling the gap in relevant publications in this field and providing a new perspective for understanding the status of current research and future research hotspots. However, the present study manually screened the publications, which may have resulted in bias. In addition, adding more synonyms to the retrieval queries may lead to a sufficient sample size and more reliable conclusions.

## Conclusions

We reviewed 1490 publications by 590 authors from 409 institutions in 77 countries. The study period was from 2008 to 2023, and the total number of publications showed an upward trend. The United States, China, and the United Kingdom are the leading countries. Chaudhuri Kallol Ray is a leader in this field. University College London (UCL) is the most prolific institution. “Movement Disorders” is the most influential journal. Daytime sleepiness is the subtype that has received the most attention. “quality of life” and “circadian rhythm” have long been the main research topics in this field. Sleep quality, biomarkers, and neurodegeneration may become future research hotspots.
